# Studies on Pharmacokinetic Drug Interaction Potential of Vinpocetine

**DOI:** 10.3390/medicines2020093

**Published:** 2015-06-05

**Authors:** Vamshi K. Manda, Bharathi Avula, Olivia R. Dale, Amar G. Chittiboyina, Ikhlas A. Khan, Larry A. Walker, Shabana I. Khan

**Affiliations:** 1 National Center for Natural Products Research, School of Pharmacy, The University of Mississippi, University, MS 38677, USA; E-Mails: vmanda@olemiss.edu (V.K.M.); bavula@olemiss.edu (B.A.); ordale@olemiss.edu (O.R.D.); amar@olemiss.edu (A.G.C.); ikhan@olemiss.edu (I.A.K.); lwalker@olemiss.edu (L.A.W.); 2 Department of BioMolecular Sciences, School of Pharmacy, The University of Mississippi, University, MS 38677, USA; 3 Department of Pharmacognosy, College of Pharmacy, King Saud University, Riyadh 12372, Saudi Arabia

**Keywords:** vinpocetine, vincamine, pharmacokinetic drug interactions, PXR, CYP450 enzymes, P-gp

## Abstract

**Background:**

Vinpocetine, a semi-synthetic derivative of vincamine, is a popular dietary supplement used for the treatment of several central nervous system related disorders. Despite its wide use, no pharmacokinetic drug interaction studies are reported in the literature. Due to increasing use of dietary supplements in combination with conventional drugs, the risk of adverse effects is on the rise. As a preliminary step to predict a possibility of drug interaction during concomitant use of vinpocetine and conventional drugs, this study was carried out to evaluate the effects of vinpocetine on three main regulators of pharmacokinetic drug interactions namely, cytochromes P450 (CYPs), P-glycoprotein (P-gp), and Pregnane X receptor (PXR).

**Methods:**

Inhibition of CYPs was evaluated by employing recombinant enzymes. The inhibition of P-gp was determined by calcein-AM uptake method in transfected and wild type MDCKII cells. Modulation of PXR activity was monitored through a reporter gene assay in HepG2 cells.

**Results:**

Vinpocetine showed a strong inhibition of P-gp (EC_50_ 8 μM) and a moderate inhibition of recombinant CYP3A4 and CYP2D6 (IC_50_ 2.8 and 6.5 μM) with no activity towards CYP2C9, CYP2C19 and CYP1A2 enzymes. In HLM, competitive inhibition of CYP3A4 (IC_50_ 54 and K_i_ 19 μM) and non-competitive inhibition of CYP2D6 (IC_50_ 19 and K_i_ 26 μM) was observed. Activation of PXR was observed only at the highest tested concentration of vinpocetine (30 μM) while lower doses were ineffective.

**Conclusion:**

Strong inhibition of P-gp by vinpocetine is indicative of a possibility of drug interactions by altering the pharmacokinetics of drugs, which are the substrates of P-gp. However, the effects on CYPs and PXR indicate that vinpocetine may not affect CYP-mediated metabolism of drugs, as the inhibitory concentrations are much greater than the expected plasma concentrations in humans.

## 1. Introduction

**Figure 1:**
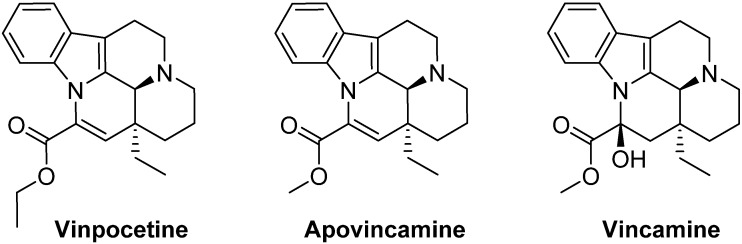
Chemical structures of vinpocetine, apovincamine and vincamine.

Vinpocetine is a semi-synthetic derivative of a natural alkaloid vincamine, present in the leaves of *Vinca minor* (lesser periwinkle) plant. Chemically, it is known as ethyl apovincaminate (Figure 1). It is commonly sold as a prescription medication in many European countries and Japan, under the brand name of Cavinton or Intelectol [1]. However, in the US, and all other countries, it is sold as a dietary supplement and is also a component of many products with muscle building and weight-loss claims. Commercially, it is primarily marketed as a neuroprotective and memory-enhancing supplement. Vinpocetine has been mainly implicated in clinical and pre-clinical studies for the treatment of several Central Nervous System (CNS) disorders, such as cerebral ischemia, epilepsy, Alzheimer’s disease, and Parkinson’s disease [2,3]. In addition to this, vinpocetine has been reported to show potential in the treatment of several inflammatory disorders, stress, glaucoma, and gastric disorders [4,5]. Vinpocetine readily crosses the blood brain barrier (BBB) to enter brain tissue [6]. It is considered to be a safe compound with no serious side effects reported in humans when administered as a single agent [7].

The risk of potential adverse drug interactions is on the rise with an increasing global trend in the combined use of conventional drugs and non-prescribed dietary supplements [8]. Many of the clinically reported drug interactions are mediated by pharmacokinetic and pharmacodyanmic alterations. Pharmacokinetic drug interactions are caused by changes in the activity or expression of drug metabolizing enzymes, efflux transporters, or nuclear receptors causing an increase or decrease in the concentration of the drug in plasma. Further, preclinical and clinical studies have identified that Cytochromes P450 (CYP), P-glycoprotein (P-gp), and Pregnane X Receptor (PXR) are the major contributors causing pharmacokinetic interactions [9]. Consequently, agents which modulate these regulatory proteins have high potential to cause pharmacokinetic drug interactions. Pharmacodynamic drug interactions result when one drug alters the pharmacological effects (additive or antagonistic) of another concomitantly taken drug. While the pharmacodynamic interactions of vinpocetine have been reported, where it was shown to enhance the effects of anticoagulant medications (warfarin) [10], no literature is available regarding its potential to cause pharmacokinetic drug interactions. Given the global use of vinpocetine as a dietary supplement and its high therapeutic potential, it is imperative to identify such pharmacokinetic interactions to avoid unwanted adverse effects when taken concomitantly with other medications. Accordingly, our current study is focused on studying the modulatory effects of vinpocetine on major drug metabolizing enzymes (CYP 3A4, 2D6, 2C9, 2C19 and 1A2), efflux transporter (P-gp), and nuclear receptor (PXR) using *in vitro* assays. The inhibition of CYPs was determined by using recombinant enzymes and specific fluorescent substrates. Calcein-AM uptake in hMDR1-MDCK-II and MDCK-II cells was determined to study the P-gp inhibition. A reporter gene assay in HepG2 cells was used to study the modulation of PXR activity.

## 2. Experimental

Mixed gender pooled human liver microsomes (HLM) prepared from 25 individual donors (protein concentration 20 mg/mL and total P450 concentration ≥0.320 nmol/mg) were obtained from Corning Life Sciences (Cambridge, MA, USAMDCK-II (parental) and hMDR1-MDCK-II (transfected) cell lines were a gift from Dr. Gottesman (NIH, Bethesda, USA). Dulbecco’s Modified Eagle Medium (DMEM), Minimal Essential Medium (MEM), Hanks Balanced Salt Solution (HBSS), HEPES, Trypsin EDTA, Penicillin-streptomycin and Sodium Pyruvate were from GIBCO BRL (Invitrogen Corp., Grand Island, NY, USA). Fetal bovine serum (FBS) was from Hyclone Lab Inc. (Logan, UT, USA). CYP1A2/3-Cyano-7-Ethoxycoumarin (*CEC*), CYP3A4/7-benzyloxy-4-trifluoromethylcoumarin (BFC), CYP2D6/3-[2-(*N*,*N*-diethyl-*N*-methylammonium)-ethyl]-7-methoxy-4-methylcoumarin (AMMC), CYP2C19/3-Cyano-7-Hydroxycoumarin (CHC), and CYP2C9/CEC high throughput inhibitor screening kits were from BD Gentest (Woburn, MA, USA). Transwell^®^ plates (12 mm diameter, 0.4 µM pore size) were from Costar Corp. (Cambridge, MA, USA). Troleandomycin was from Santa Cruz Biotechnology, Inc. (Dallas, TX, USA). Testosterone, 6β-hydroxytestosterone, vinpocetine, magnesium chloride, corticosterone and all other chemicals were purchased from Sigma-Aldrich (St Louis, MO, USA). NADPH regeneration solution A and B were purchased from Corning Discovery Labware (Woburn, MA, USA). HPLC grade acetonitrile, methanol and formic acid were from Fisher Scientific (Waltham, MA, USA). The purity of the test compounds and positive controls used in the study was ≥95%. The final concentrations of DMSO and methanol did not exceed 0.1% and 0.5%, respectively, in all the experiments.

## 3. Culture of hMDR1-MDCK-II, MDCK-II and HepG2 Cells

Parental and transfected MDCK-II cells were cultured in Dulbecco’s Modified Eagle’s Medium (DMEM) supplemented with 10% fetal bovine serum, 1% non-essential amino acids, 1% l-glutamine, 100 U/mL penicillin-G, and 100 μg/mL streptomycin at 37 °C, 95% relative humidity, and 5% CO_2_. HepG2 cells were cultured in DMEM/F12 medium supplemented with 10% fetal bovine serum, 2.4 g/L sodium bicarbonate, 100 U/mL penicillin-G, and 100 μg/mL streptomycin at 37 °C, 95% relative humidity, and 5% CO_2_.

## 4. Reversible Inhibition (Co-Incubation Assay) and Time Dependent Inhibition (TDI/pre-Incubation Assay) of CYPs

The assay conditions for determining reversible and time dependent inhibition of CYPs were similar as reported earlier [11,12]. Test samples or positive controls were serially diluted in a solution (100 µL) of cofactors mix, control protein (0.05 mg of protein/mL) and Glucose-6-phosphate dehydrogenase (G6PDH) to achieve six concentrations (100–0.4 µM). To account for any inherent fluorescence, the plates were incubated at 37 °C for 10 min and fluorescence readings were taken. Enzyme substrate mixture (100 µL) was added to initiate the reaction and the plates were further incubated for 15 (CYP1A2), 30 (CYP3A4, 2C9 and 2C19) or 45 min (CYP2D6). The reaction was stopped by the addition of 75 µL of acetonitrile/0.5M Tris base (80:20). Fluorescence was measured on Spectramax M5 plate reader (Molecular Devices, Sunnyvale, CA, USA) at specified excitation and emission wavelengths for each substrate. IC_50_ values (co-incubation assay) were obtained from concentration-response curves generated by plotting log concentration *versus* % control.

For TDI activity of vinpocetine, the initial incubation mixture (180–190 µL), consisting of test sample or positive control, recombinant enzyme, control protein (0.05 mg of protein/mL), cofactor mix, G6PDH, and 50 mM potassium phosphate buffer (pH 7.4), was pre-incubated for 30 min followed by addition of respective fluorescent substrates (10–20 µL) and further incubation for 15 (CYP1A2), 30 (CYP3A4) or 45 (CYP2D6) min. The reaction was terminated and fluorescence was measured as described above. IC_50_ values (pre-incubation assay) were obtained as above. The IC_50_ shift fold was calculated as the ratio of IC_50_ (co-incubation)/IC_50_ (pre-incubation).

## 5. Reversible Inhibition and Enzyme Kinetics of CYP3A4 and CYP2D6 in Pooled HLM

The reversible inhibition of CYP3A4 and CYP2D6 was determined in terms of the metabolic conversion of testosterone to 6β-hydroxytestosterone and dextromethorphan to dextrorphan, respectively, according to a procedure described previously [13]. The incubation mixture (250 µL) consisted of NADPH regeneration system (NADP^+^, glucose-6-phosphate, and G6PDH) in 0.1M potassium phosphate buffer (pH 7.4). Vinpocetine or positive controls (100–0.05 µM) were added to the reaction mixture and pre-warmed for 10 min. The reaction was initiated by the addition of HLM (0.6 mg/mL protein). After further incubation of 30 min (for CYP3A4) or 20 min (for CYP2D6) at 37 °C, reaction was terminated by addition of ice-cold acetonitrile containing 5 μL corticosterone (internal standard for CYP3A4, 20 µM) or 5 μL niflumic acid (internal standard for CYP2D6, 1 µM). The mixture was centrifuged at 10,000 rpm for 5 min and the supernatant (200 μL) was transferred into vials for HPLC (CYP3A4) or UHPLC (CYP2D6) analysis. IC_50_ values were obtained from concentration-response curves generated by plotting log concentration *versus* % of control by using GraphPad Prism 6 (La Jolla, CA, USA).

The type of inhibition (competitive or non-competitive) was predicted by Lineweaver-Burk Plot (velocity^−1^ *vs.* substrate^−1^). Apparent inhibition constant (K*_i_*) was calculated from the x-intercept of the Dixon plot (slope of the primary plot *vs*. inhibitor concentration), which was fitted by liner regression using GraphPad Prism 6 (La Jolla, CA, USA).

## 6. PXR Modulation

The pSG5-hPXR expression vector was provided generously by Dr. Steven Kliewer (University of Texas Southwestern Medical Center, Dallas, TX, USA) [14] and the reporter plasmid CYP3A4-PXR response element (PXRE)-LUC (containing the proximal 0/–362 and distal 7208/7797 PXRE regions fused upstream of luciferase) [15] was a kind gift from Dr. Christopher Liddle (University of Sydney, Westmead, Australia). The modulation of PXR activity by vinpocetine was determined in HepG2 cells transiently transfected with pSG5-PXR and PCR5 plasmid DNA (25 µg each) by electroporation at 180 V, 1 pulse for 70 ms as described earlier [11,16]. The cells were plated in 96-well plates at a density of 50,000 cells per well and incubated for 24 h. Test samples and drug controls were added at various concentrations (30–1 µM). After treatment for 24 h, the media was aspirated from the cells and 40 μL of luciferase reagent (Promega Corporation, Madison, WI, USA) was added to each well and luminescence was measured on Spectramax M5 plate reader (Molecular Devices, Sunnyvale, CA, USA). The fold induction in luciferase activity in the treated cells was calculated in comparison to vehicle treated cells.

## 7. P-gp Inhibition

The assay was performed by following reported method [17]. MDCK-II and hMDR1-MDCK-II cells were seeded in 96-well plates at 70,000 cells/well in 200 µL of culture medium. The medium was changed at 24 h after seeding and the assay was performed 48 h later. Test samples and positive control at various concentrations (100–0.4 µM) were added to the cells in 50 µL of transport buffer and incubated at 37 °C for 10 min. Calcein-AM, a fluorescent P-gp substrate (1 µM), was added and the plates were immediately placed on Spectramax plate reader and fluorescence was read up to 1 h at 15-min intervals at excitation and emission wavelengths of 485 and 530 nm, respectively. The % increase in calcein-AM uptake was calculated as described earlier [18]. The EC_50_ value, defined as the concentration that caused an increase of 50% in calcein-AM uptake, was obtained from dose curves generated by plotting % increase in calcein-AM uptake *versus* log concentration using GraphPad Prism.

## 8. Analytical Methods

Testosterone and 6β-hydroxytestosterone were quantified according to the previously described method [19]. The HPLC system consisted of Waters model 6000A pumps, U6K injector, 680 automated gradient controller, 996 PDA and Empower 2 software (Waters Corp., Milford, MA, USA). A Gemini C18 column (150 × 4.6 mm; 5 µm particle size) from Phenomenex (Torrance, CA, USA) was used as stationary phase and temperature was maintained at 30 °C. The column was equipped with a 2 cm LC-18 guard column (Phenomenex, Torrance, CA, USA). The mobile phase consisted of water (C) and acetonitrile (D) both containing 0.1% formic acid at a flow rate of 1.0 mL/min. The limit of detection (LOD) and limit of quantification (LOQ) for testosterone and 6β-hydroxytestosterone were 0.5 µg/mL and 0.1 µg/mL, respectively.

The quantification of dextromethorphan, dextrorphan and niflumic acid (internal standard) was done by using Agilent LC-quadrupole time-of-flight (QToF) (model 6230)-mass spectrometry (Agilent Technologies, Palo Alto, CA, USA). The UHPLC system consisted of a binary pump, autosampler, degasser, and a column compartment. The mass detector was equipped with an electron spray ionization interface, which was connected to Agilent MassHunter Work Station (B.05.00). Dextromethorphan and dextrorphan were analyzed using an Agilent Zorbax SB-C8 RRHD column (100 mm × 2.1 mm I.D., 1.8 µm). A column temperature of 35 °C and sample temperature of 15 °C were maintained during analysis. The mobile phase consisted of water (A) and Acetonitrile (B), both containing 0.1% formic acid, which were applied in the following linear gradient elution: 0 min, 80%A: 20%B in next 10 min to 30%A: 70%B and in next 2 min to 100% B, at a flow rate of 0.2 mL/min. Separation was followed by a 2 min washing procedure with 100% B and re-equilibration period of 3.0 min. The total run time for analysis was 10 min. The injection volume was 2 µL. Compounds were confirmed in positive ion mode with *m*/*z* = 272.1998 [M + H]^+^, 258.1844 [M + H]^+^, and 283.0676 [M + H]^+^ for dextromethorphan, dextrorphan, and niflumic acid, respectively. The LOD and LOQ of dextromethorph and dextrorphan, were 10 ng/mL and 5 ng/mL, respectively.

## 9. Statistical Methods

The data were analyzed by one way ANOVA, followed by Dunnett’s multiple comparison tests using GraphPad Prism Version 6, (San Diego, CA, USA). *p* < 0.05 was considered to be statistically significant.

## 10. Results and Discussion

Pharmacokinetic drug-drug interactions have led to withdrawal of prescription medications like astemizole, mibefradil, and terfenadine due to life threatening side effects. Follow-up studies have shown that these drug interactions were primarily due to inhibition of CYP enzymes. As a result, the plasma concentrations in humans reached toxic levels when taken in combination with drugs (erythromycin, ketoconazole, and cimetidine) that were potent inhibitors of CYP3A4 [20]. St. John’s wort is a well-documented example of dietary supplement causing therapeutic failure primarily due to the up-regulation of PXR by its constituents (hyperforin and hypericin) and thereby decreasing the plasma concentrations of drugs like indinavir, digoxin, cyclosporin, *etc.* [21]. Furthermore, dietary supplements rich in flavonoids like quercetin have been shown to increase the oral absorption of drugs like doxorubicin by inhibition of intestinal P-gp *in vivo* [22]. Consequently, Food and Drug Administration (FDA) has recommended preliminary *in vitro* studies to identify compounds that may have potential to cause drug interactions. These studies are primarily focused on evaluating the modulatory effects of an investigational compound/drug on CYPs, P-gp or PXR [23].

**Figure 2:**
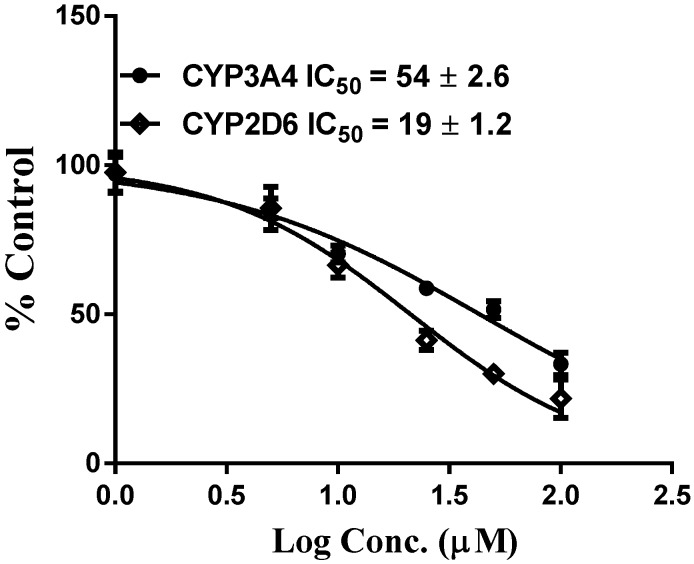
Concentration response profiles and IC_50_ values of reversible inhibition of CYP3A4 and CYP2D6 in HLM by vinpocetine. Each point is the mean ± SD of three independent incubations in HLM.

**Figure 3:**
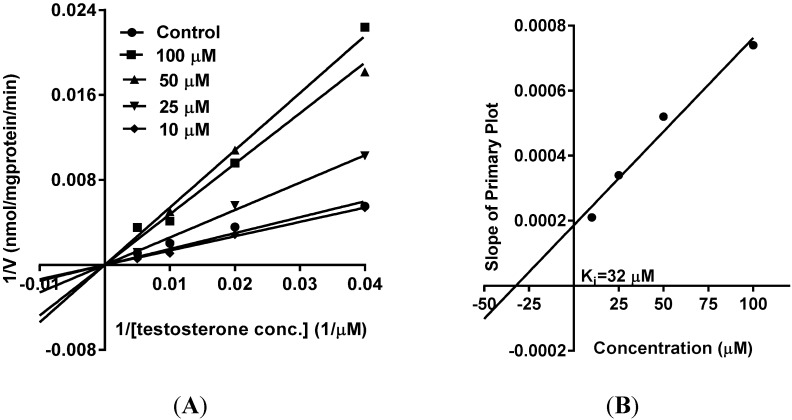
(**A**) Primary Lineweaver-Burk Plot of CYP3A4 inhibition (competitive) by vinpocetine in HLM; (**B**) Inhibition constant (K_i_) was determined by plotting the slope of the Primary Lineweaver-Burk Plot *versus* the concentration of vinpocetine. The data are represented as mean ± SD of triplicate incubations in HLM.

**Figure 4:**
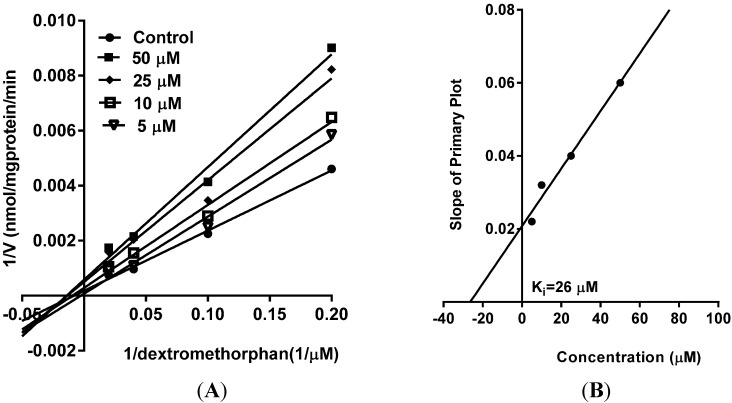
(**A**) Primary Lineweaver-Burk Plot of CYP2D6 inhibition (competitive) by vinpocetine in HLM; (**B**) Inhibition constant (K_i_) was determined by plotting the slope of the Primary Lineweaver-Burk Plot *versus* the concentration of vinpocetine. The data are represented as mean ± SD of triplicate incubations in HLM.

As part of our ongoing studies on safety of herbal dietary supplements, this study was carried out to evaluate the effects of vinpoectine on CYPs, P-gp and PXR activity and thereby determine its pharmacokinetic drug interaction potential. Using recombinant CYPs and specific fluorescent substrates, reversible and TDI of CYPs by vinpocetine was evaluated. It showed reversible inhibition of both CYP3A4 and CYP2D6 enzymes with IC_50_ values of 2.80 ± 0.98 and 6.5 ± 1.1 µM, respectively (Table 1 and Table 2). No inhibition was seen with the other CYPs tested (CYP2C9 and CYP2C19) (data not shown). However, these IC_50_ values were significantly higher than the control drugs, ketoconazole (IC_50_ = 0.04 µM) and quinidine (IC_50_ = 0.05 µM) (Table 1 and Table 2), suggesting a moderate inhibitory activity of vinpocetine. It is considered that compounds which show an IC_50_ shift ratio of greater than 1.5 have potential to exhibit TDI. Based on these criteria, no TDI of vinpocetine was observed with either CYP3A4 or CYP2D6 (IC_50_ shift ratio 0.54 and 0.17, respectively), as shown in Table 1 and Table 2. These data indicate that the inhibition of CYP3A4 and CYP2D6 is primarily mediated by vinpocetine with no formation of reactive metabolites during the pre-incubation time. The IC_50_ shift fold ratios of the positive controls for TDI, troleandomycin (5.9) and paroxetine (5.4) (Table 1 and Table 2) were similar to the published literature values [24]. Based on the recombinant CYP inhibition results, we further evaluated the inhibitory activity of vinpocetine on CYP3A4 and CYP2D6 in HLM using testosterone and dextromethorphan as probe substrates. As seen in Figure 2, a very weak inhibition of CYP3A4 and CYP2D6 was observed with IC_50_ values of 54 ± 2 and 19 ± 1 µM, respectively. The IC_50_ value with recombinant CYP3A4 enzymes was almost 19-fold lower than with HLM. These differences can be attributed to the differences in substrates (BFC in recombinant CYP and testosterone in HLM) used in the assays. The catalytic site of CYP3A4 enzyme has been shown to possess two different binding sites and thereby structurally diverse compounds are known to bind to the enzyme [25]. Also, BFC and testosterone have been shown to bind to different sites of the enzyme [26], which may also explain the differences in the inhibition profiles. Additional factors such as non-specific binding to other CYP enzymes in HLM and competition from long-chain fatty acids may also be responsible for the differences in the inhibition profile of vinpocetine. Furthermore, kinetic analysis in HLM showed competitive inhibition of testosterone metabolism (mediated by CYP3A4) by vinpocetine with a K_i_ value of 32 µM, as shown in Figure 3. Non-competitive inhibition of dextromethorphan metabolism (mediated by CYP2D6) was seen with a K_i_ value of 26 µM (Figure 4). It should also be noted that vinpocetine has been shown to have a very low oral bioavailability (6%) in humans with maximum plasma concentration (Cmax) value of 60 ng/mL [27]. Hence, it is highly unlikely that the inhibitory concentrations (IC_50_ and K_i_) observed in this study will be achieved *in vivo* to inhibit CYP3A4 and CYP2D6 and thus the possibility of CYP mediated drug interaction seems to be remote.

**Table 1:** IC_50_ values of CYP3A4 inhibition. The data are represented as mean ± SD of three independent experiments.

Compound	CYP3A4 (recombinant)		
	IC_50_ (µM)	IC_50_ (µM)	IC_50_
	Co-incubation	Pre-incubation	Shift (Fold)
Vinpocetine	2.8 ± 0.98	5.1 ± 0.1	0.54
Ketoconazole	0.04 ± 0.001	0.05 ± 0.002	0.80
Troleandomycin	2.5 ± 0.8	0.42 ± 0.05	5.95

**Table 2:** IC_50_ values of CYP2D6 inhibition. The data are represented as mean ± SD of three independent experiments.

Compound	CYP2D6 (recombinant)		
	IC_50_ (µM)	IC_50_ (µM)	IC_50_
	Co-incubation	Pre-incubation	Shift (Fold)
Vinpocetine	6.5 ± 1.1	37 ± 1.6	0.17
Quinidine	0.05 ± 0.001	0.08 ± 0.002	0.62
Paroxetine	3.4 ± 0.9	0.62 ± 0.03	5.48

**Figure 5:**
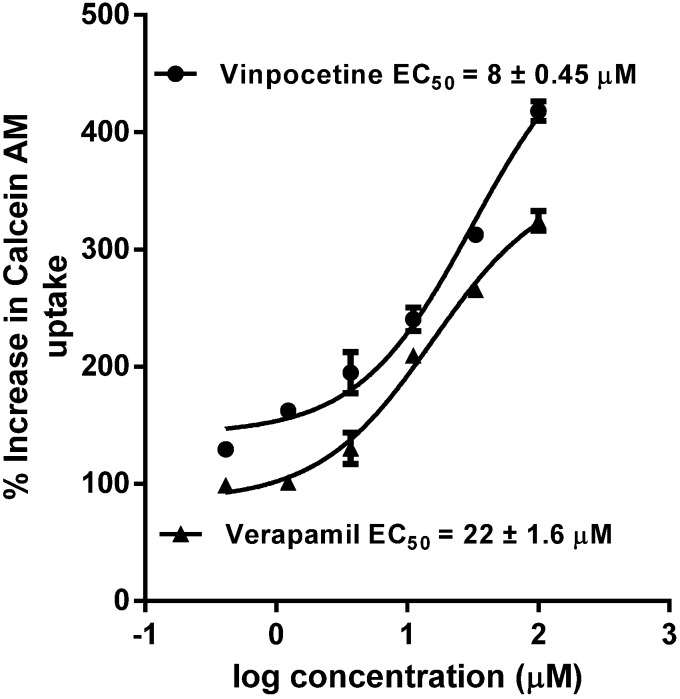
Concentration response curve and EC_50_ values of P-gp inhibition by vinpocetine and verapamil (positive control). Each point is the mean ± SD of three independent experiments.

The presence of P-gp on different tissues and its ability to efflux diverse range of substrates has made it the primary transporter to regulate the pharmacokinetic properties of xenobiotics [28]. Calcein-AM uptake in MDCKII and hMDR1-MDCKII cells was used to quantify P-gp inhibitory activity of vinpocetine. It showed potent inhibition of P-gp with an IC_50_ value of 8.0 ± 0.45 µM, which was 2.7-fold lower than positive control verapamil (22.0 ± 0.6 µM), as shown in Figure 5. This potent inhibition of P-gp by vinpocetine may be attributed due to the presence of ester functional group in its structure. The presence of ester group has shown to increase the affinity of a compound towards P-gp catalytic site [29]. Furthermore, several ester containing natural compounds (euphoportlandols, A and B, tropane esters, and pervilleines) have been shown to be strong inhibitors of P-gp. Also, compounds which show IC_50_ values below 10 µM in Calcein-AM transport assay are further recommended for *in vivo* studies [30]. While this P-gp inhibition may lead to drug interactions, it could also be beneficial in increasing the uptake of concomitantly taken P-gp substrate drugs with low bioavailability and blood brain barrier (BBB) penetration in combination with vinpocetine.

**Figure 6:**
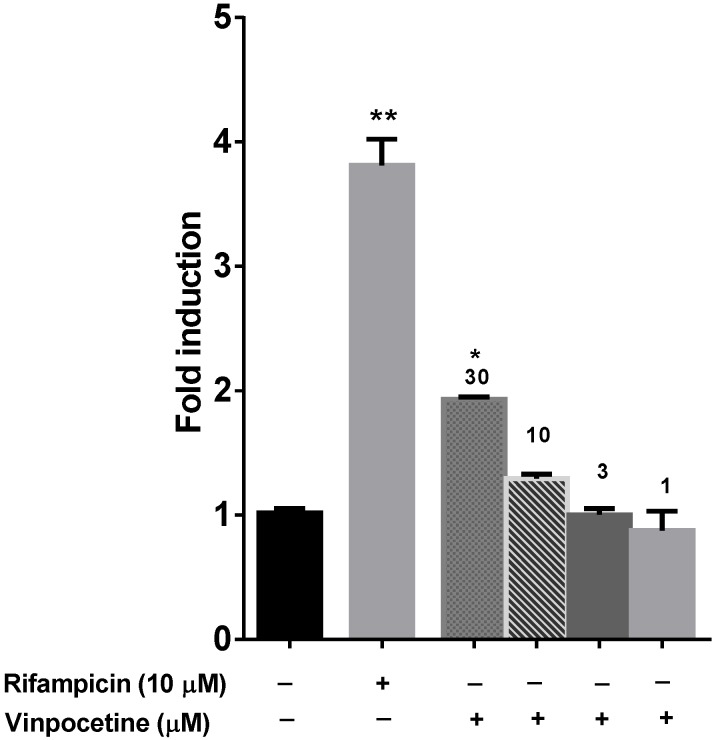
Induction of PXR activity by vinpocetine and rifampicin at indicated concentrations. * *p* < 0.05, and ** *p* < 0.01 determined by One way ANOVA, followed by Dunnett’s multiple comparison tests. The data are represented as mean ± SEM of duplicate experiments.

The nuclear hormone receptor PXR is a known transcriptional regulator of several CYP enzymes (CYP3A4, CYP1A2, CYP2C9, and CYP2C19) and efflux transporters (P-gp) [31]. Since activation of PXR was observed only at high concentration of vinpocetine (30 µM), as shown in Figure 6, possibility of drug interactions due to modulation of PXR are very remote because it is highly unlikely that these concentrations could be achieved *in vivo*.

## 11. Conclusions

To our knowledge, this is the first preliminary *in vitro* report studying the pharmacokinetic drug interaction potential of vinpocetine. CYP3A4 and CYP2D6 inhibitory activities by vinpocetine were moderate in recombinant enzymes; however, weak inhibition was seen with HLM. The P-gp inhibition was stronger than verapamil, implicating the possibility of potential drug interactions with other drugs that are substrates for P-gp. Further studies are needed to see if the bioavailability and distribution of P-gp substrates are altered *in vivo* in presence of vinpocetine. No significant activation of PXR was seen with vinpocetine.
